# Case Report: A rare case of fucosidosis caused by a novel homozygous pathogenic variant in the FUCA1 gene within a 17.2 Mb region of homozygosity

**DOI:** 10.3389/fmed.2026.1573922

**Published:** 2026-06-30

**Authors:** Hua Wang, Weirong Xing

**Affiliations:** 1 Division of Genetics, Department of Pediatrics, Loma Linda University School of Medicine, Loma Linda, CA, United States; 2 Department of Medicine, Loma Linda University School of Medicine, Loma Linda, CA, United States; 3 Musculoskeletal Disease Center, VA Loma Linda Healthcare System, Loma Linda, CA, United States

**Keywords:** FUCA1 gene, fucosidosis, lysosomal storage disease, novel homozygous pathogenic variant, region of homozygosity

## Abstract

Fucosidosis is a rare lysosomal storage disorder caused by mutations in the FUCA1 gene, leading to deficient α-L-fucosidase activity and the accumulation of fucosylated substrates. The clinical spectrum varies widely, encompassing neurological regression, coarse facial features, angiokeratomas, hepatosplenomegaly, and systemic involvement. Here, we report a case of fucosidosis in a patient presenting with prominent progressive neurological symptoms and atypical angiokeratoma-like skin lesions, but notably absent hepatosplenomegaly or cardiac involvement. Additional features included recurrent respiratory illnesses. Genetic analysis identified a novel homozygous nonsense mutation, c.557 T > A (p.Leu186*), in the FUCA1 gene located within a 17.2 Mb region of homozygosity at 1p36.21p35.2. This variant truncates the catalytic domain and expands the FUCA1 mutation spectrum. The case highlights the disorder’s phenotypic heterogeneity and the importance of early genetic diagnosis to enable timely interventions such as hematopoietic stem cell transplantation (HSCT).

## Introduction

Fucosidosis (OMIM #230000) is an autosomal recessive lysosomal storage disorder (LSD) caused by biallelic pathogenic variants in the *FUCA1* gene (OMIM #612280), located on chromosome 1p36.11 (1). This gene encodes the enzyme α-L-fucosidase (EC 3.2.1.51), a homotetramer responsible for the hydrolysis of terminal α-L-fucoside linkages in glycosphingolipids and glycoproteins. Deficiency of this enzyme results in the progressive accumulation of fucose-containing glycolipids and glycoproteins in lysosomes, leading to a multisystem disorder characterized by neurodegeneration, developmental delay, progressive motor impairment, intellectual disability, and coarse facial features (2, 3). Additional clinical manifestations may include growth retardation, recurrent respiratory infections, angiokeratomas, and skeletal abnormalities (4). The phenotypic spectrum of fucosidosis varies widely, with disease severity influenced by the nature of the mutations in FUCA1 gene. Historically, the disorder has been categorized into two subtypes: Type I, which presents early in infancy with severe symptoms and rapid progression, and Type II, which is associated with later onset, milder symptoms, and longer survival (5). Recent studies, however, advocate for a continuum of disease severity rather than discrete subtypes.

The estimated incidence of fucosidosis is less than 1 in 200,000 live births (6), classifying it as an ultra-rare disorder. While the condition has been reported globally, certain populations exhibit higher prevalence due to founder effects and genetic isolation. These include regions of southern Italy, the Hispanic American populations in New Mexico and Colorado, and Cuba, as well as other areas with limited genetic diversity (2). The distribution of cases reflects both the low general incidence and the impact of local founder mutations, underscoring the importance of understanding population genetics in the context of rare diseases. Despite its rarity, fucosidosis is likely underdiagnosed in some regions due to its overlapping features with other lysosomal storage disorders and limited access to diagnostic testing.

To date, about 37 pathogenic variants in the *FUCA1* gene have been identified, encompassing a wide range of mutation types, including missense, nonsense, frameshift, splice-site alterations, and large deletions (HGMD 2024). These mutations disrupt the normal synthesis, folding, or activity of α-L-fucosidase, leading to varying degrees of enzyme deficiency and clinical manifestations. Globally, fewer than 130 cases of fucosidosis have been reported in the literature, with the majority described in pediatric populations (2). Here, we reported a newly identified case of fucosidosis originated from a Hispanic heritage family.

## Case description

We present a male patient of Mexican descent, first evaluated at age two in 2019. Born at 36 weeks via spontaneous vaginal delivery to a G2P2 mother, his neonatal course was notable for jaundice requiring phototherapy. Early developmental delays included absent facial expressions and no rolling by 6 months. After relocating to Mexico at 8 months, he showed profound delays, with sitting at 14 months, crawling at 16 months, no verbal speech, and no independent walking. He was hospitalized for hypoxia due to respiratory illness upon returning to the U.S. in 2019, leading to a genetics referral.

Initial genetics evaluation revealed a nonverbal child with significant motor impairments. The patient was able to sit but could not stand or walk independently. Dysmorphic features were subtle, with no coarse facies, hepatosplenomegaly, or hirsutism. Musculoskeletal findings included lumbar kyphosis, a gibbus deformity, and spasticity in the lower extremities with in-toeing foot abnormalities. Mongolian spots were noted on the back.

Subsequent brain MRI demonstrated diffuse white matter signal abnormalities and bilateral globus pallidus involvement, suggestive of a metabolic disorder such as leukodystrophy or mucopolysaccharidosis (MPS) (see Figure 1A). A thoracolumbar MRI revealed hypoplastic vertebral bodies with anterior beaking and mild kyphosis, features commonly associated with severe forms of MPS (see Figure 1B). An EEG demonstrated nonspecific findings of cerebral dysfunction without evidence of seizure activity. Despite these findings, MPS enzyme and urine screening results were unremarkable, ruling out common lysosomal storage disorders. Ophthalmologic examination revealed myopia with astigmatism but no evidence of storage-related ocular abnormalities. Further genetic testing of chromosomal microarray analysis (Mayo clinic laboratory) identified a 17.2 MB region of homozygosity (ROH) at arr[hg19] 1p36.21p35.2(14,704,440–31,922,034)x2 hmz raising the possibility of an autosomal recessive disorder due to a homozygous mutation in this region and/or uniparental disomy (UPD) of chromosome 1. Whole-exome sequencing was initiated but could not be completed due to the family’s relocation back to Mexico later in 2019 and subsequent disruptions during the COVID-19 pandemic.

**Figure 1:**
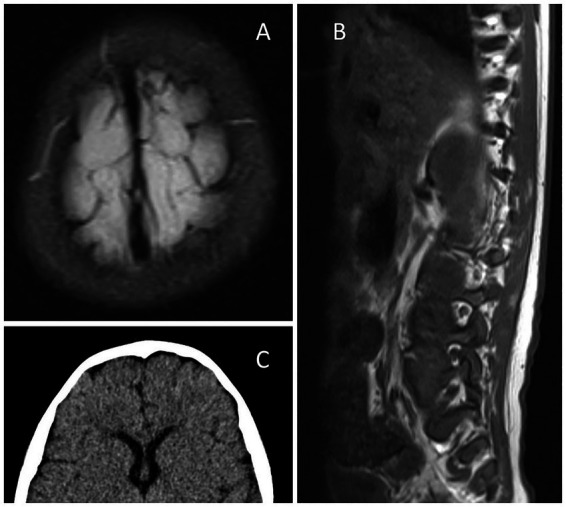
**(A)** Brain MRI demonstrated diffuse white matter signal abnormalities and bilateral globus pallidus involvement, suggestive of a metabolic disorder such as leukodystrophy or mucopolysaccharidosis; **(B)** thoracolumbar MRI revealed hypoplastic vertebral bodies with anterior beaking and mild kyphosis, features commonly associated with severe forms of MPS; **(C)** CT imaging study of the head confirmed persistent hypodensities in the periventricular white matter and bilateral globus pallidus, consistent with leukodystrophy.

In early 2023, at age six, the patient returned to the U.S. and was hospitalized in later that year with spastic quadriplegic cerebral palsy, neurogenic bladder, speech delay, fever, oral blisters, and cold extremities. A genetics consult was requested. During his time in Mexico, the patient had frequent hospitalizations for respiratory infections. Developmentally, he crawled at 16 months but lost this ability by age six. His hand clamping regressed into finger contractures, and leg stiffness worsened. Exam revealed coarse facial features, thick eyebrows, high nasal bridge, oral blisters, micrognathia (Figure 2A), muscular atrophy, abnormal lower limb posture secondary to hypertonia (Figure 2B), and red-purple, pinhead-sized papules on the trunk.

**Figure 2:**
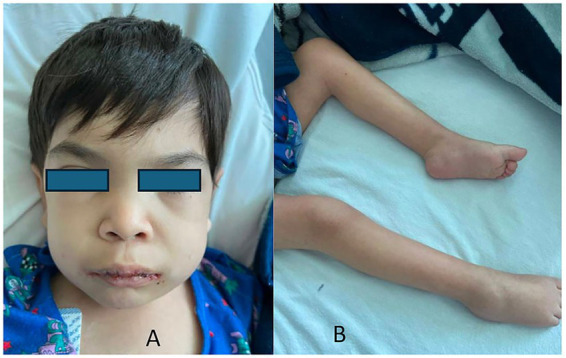
**(A)** Coarse face, thick eyebrow, high and wide nasal bridge; **(B)** Muscular atrophy and abnormal lower extremity positioning/posturing.

Further CT imaging study of the head confirmed persistent hypodensities in the periventricular white matter and bilateral globus pallidus, consistent with leukodystrophy (see Figure 1C). Viral studies revealed HSV-1 infection, and the patient was treated with intravenous acyclovir. A leukodystrophy genetic panel identified a homozygous pathogenic variant c.557T>A (p.Leu186*) in the FUCA1 gene, which is located in the previously identified ROH. Further lysosomal enzyme testing using a whole blood panel confirmed the diagnostic impression, revealing a complete absence of α-L-fucosidase activity (0 nmol/h/mg; reference range: 18.1–142.8 nmol/h/mg), while neighboring lysosomal enzyme activities remained entirely within normal physiological ranges.

Parental genetic analysis: Following the identification of the proband’s genotype, parental segregation analysis was pursued to confirm inheritance patterns. Both parents are from Mexico, and the family reported no known history of genetic conditions or consanguinity. The mother was confirmed to be a heterozygous carrier of the c.557T>A variant. The father was unavailable for carrier testing; however, the presence of the homozygous variant in the proband within a 17.2 Mb region of homozygosity (ROH) strongly suggests biallelic inheritance from a common ancestor, increasing the likelihood of distant consanguinity despite the unremarkable family history. Genetic counseling was provided regarding recurrence risks.

The patient was discharged to a rehabilitation facility for multidisciplinary therapy. Due to dysphagia, feeding was limited to pureed foods. In 2023, he showed worsening dystonia, spasticity, coarse facial features, and skin rashes. Imaging revealed prominent renal sinus fat and heterogeneous pancreatic texture, suggesting fat deposition. Polysomnography showed mild snoring without sleep apnea, and audiometry indicated bilateral middle ear dysfunction. Despite supportive care, his condition continues to decline, consistent with progressive nature of fucosidosis.

## Clinical evolution and follow-up (2023–2026)

Following molecular diagnosis, the patient experienced progressive neurodegenerative decline, multi-system fragility, and chronic malnutrition.

- 2023: Hospitalized for viral pneumonia complicated by acute respiratory failure.
- 2024: Diagnosed with bilateral moderately severe sensorineural hearing loss (SNHL), bilateral esotropia, ocular hypertension, and early senile nuclear sclerosis. Underwent an adenoidectomy, myringotomy, and bilateral tympanostomy tube placement under general anesthesia (GA) due to chronic upper airway obstruction and recurrent otitis media.
- In 2026: Severe cachexia manifested with weight and stature Z-scores dropping below −5.05 and −5.88 SD. Severe dental disease led to left mandibular premolar cellulitis with cortical breakthrough. Severe baseline dystonia required hospitalization for full-mouth dental rehabilitation and multiple extractions under GA using specialized volatile-induction protocols.
- Later in 2026: Recurrent infections (including Influenza A, Adenovirus, and *Bordetella parapertussis*) triggered severe status dystonicus. Episodes presented as hour-long cycles of generalized rigid dystonia and autonomic color changes.

Current management: Pediatric neurology initiated acute illness-linked clonidine coverage (0.05 mg PO BID for 7 days), intranasal rescue midazolam (Valtoco 5 mg) for events exceeding 5 min, and a slow outpatient lamotrigine titration toward a 50 mg BID target. As of in 2026, the patient remains nonverbal and non-ambulatory (GMFCS Class V), dependent on an adaptive wheelchair with a custom pelvic positioning harness and an eye-gaze communication device.

## Discussion

### Fucosidosis phenotype

Fucosidosis is a progressive neurodegenerative lysosomal storage disorder characterized by neurological symptoms, including motor impairment, spasticity, seizures, and intellectual decline (4). In this case, the patient demonstrated these hallmark features. The patient’s development of spastic quadriplegic cerebral palsy, and worsening contractures align with the motor deterioration described in up to 87% of fucosidosis cases (3). Additionally, the neurological decline including dystonia, worsening leg stiffness and contractures documented in 40% of cases (7).

In this case, the patient demonstrated classical hallmark features of motor deterioration, including spastic quadriplegic cerebral palsy and progressive joint contractures, which are documented in up to 87% of affected individuals. However, his intermediate rate of neurodegenerative progression and survival into late childhood represents a phenotypic continuum between the rigid Type I and Type II classification frameworks. While his early milestones (sitting at 14 months, crawling at 16 months) were significantly delayed compared to healthy peers, they align broadly with the childhood-onset cohort variant descriptions. The absence of early, generalized cerebral or cerebellar atrophy on neuroimaging further distinguishes this intermediate clinical trajectory from rapidly devastating infantile presentations.

However, there is significant phenotypic heterogeneity across the patient population, ranging from severe infantile forms (Type I) to milder juvenile forms (Type 2) (5, 8). This patient’s disease course, with delayed developmental milestones and progressive neurological symptoms but survival into childhood, appears to represent an intermediate phenotype, reflecting the continuum often observed in clinical presentations. While the patient achieved milestones such as sitting at 14 months and crawling at 16 months, his developmental progress was markedly delayed compared to the average onset of neurological symptoms at 1.2 ± 0.8 years (3). Physical findings in fucosidosis frequently include coarse facial features, thickened lips, macroglossia, and dysostosis multiplex, along with angiokeratoma corporis diffusum in over half of cases (3, 4). In this patient, the coarse facial features, including a wide nasal bridge and micrognathia, were consistent with these findings. The red-purple skin lesions on the trunk resembled angiokeratomas, a distinguishing feature of advanced fucosidosis. However, hepatosplenomegaly, reported in 20–40% of cases (3), was notably absent in this patient, underscoring the variability in systemic involvement.

Recurrent respiratory infections, observed in up to 78% of fucosidosis cases (3) were a prominent feature in this patient’s clinical course. This ongoing susceptibility necessitated frequent hospitalizations and specific surgical interventions, including adenoidectomy and tympanostomy tube placement in 2024 to manage chronic upper airway obstruction and recurrent otitis media. Furthermore, the patient suffered from severe dental deterioration with multiple abscesses, a complication likely exacerbated by sialorrhea, mouth breathing, and limited oral care access due to severe dystonia. This history of repeated infectious episodes highlights the immunological vulnerabilities often associated with lysosomal storage disorders.

Ophthalmologic findings, such as tortuous retinal and conjunctival vessels or pigmentary retinopathy, have been reported in up to 54% of cases (9). Initially, our patient presented only with myopia and astigmatism; however, longitudinal follow-up revealed the development of bilateral ocular hypertension and lenticular opacities (nuclear sclerosis) by age eight. This evolution highlights the progressive nature of ocular involvement in fucosidosis, which may not be present in early childhood.

Neuroimaging often reveals hypomyelination with extensive, symmetric, and progressive signal abnormalities in the periventricular and subcortical white matter. T2-weighted imaging typically shows hypointense areas in the bilateral globus pallidus. In advanced stages, particularly in the type II phenotype, cerebral and cerebellar atrophy is frequently observed. Magnetic resonance spectroscopy (MRS) commonly demonstrates a reduced N-acetyl aspartate (NAA)/choline ratio, reflecting neuronal loss or dysfunction (10–13). In our patient, brain MRI revealed diffuse white matter abnormalities and bilateral globus pallidus involvement, consistent with these reported features of fucosidosis. CT imaging also showed persistent hypodensities in the periatrial white matter and globus pallidus, aligning with the leukodystrophy-like changes commonly seen in this condition. However, unlike advanced cases that often exhibit generalized cerebral and cerebellar atrophy, our patient showed no significant atrophy, suggesting an earlier stage of disease progression. These neuroimaging findings, in combination with clinical and biochemical data, play a crucial role in diagnosing fucosidosis and understanding its progression.

While this case reflects many classical features of fucosidosis, such as neurological decline, dysmorphic features, and angiokeratomas, certain aspects, including the absence of hepatosplenomegaly and initially limited ocular findings, highlight the variability in clinical manifestations. The patient’s HSV-1 infection and associated systemic symptoms represent secondary complications not directly related to lysosomal storage pathology but contributed to the complexity of disease management.

### FUCA1 gene and 1p36.21p35.2 region of homozygosity (ROH)

The FUCA1 gene, located at chr1:23,754,075–23,775,841 (GRCh37/hg19, NCBI Gene ID 251), lies within a 17.2 Mb region of homozygosity (ROH) identified by chromosomal microarray at arr[hg19] 1p36.21p35.2(14,704,440–31,922,034)x2 hmz. FUCA1 gene encodes α-L-fucosidase spans ~ 23 kb with eight exons and encodes a 461-amino acid protein comprising a signal peptide (residues 1–22), a catalytic domain (residues 35–370), and a C-terminal domain (residues 372–461). The catalytic core adopts a (β/α)₈-barrel structure essential for hydrolytic activity (14–16). The active site includes Asp276, a key acid/base residue (see Figures 3A,B) enabling proton transfer during hydrolysis, distinguishing FucA1 from other glycosidases that use glutamate. To date, the HGMD Professional database (2023.4) reports 37 distinct pathogenic variants in *FUCA1*, including missense/nonsense mutations (18), splicing variants (4), small deletions (9), small insertions (1), gross deletions (4), and one gross insertion/duplication. Pathogenic variants disrupt the enzymatic activity of α-L-fucosidase, causing lysosomal accumulation of fucose-containing glycolipids and glycoproteins with cross-reacting immunologic material (CRIM) levels below 6% of normal, suggesting rapid degradation and instability of mutant fucosidase proteins (1, 3, 17, 18).

**Figure 3:**
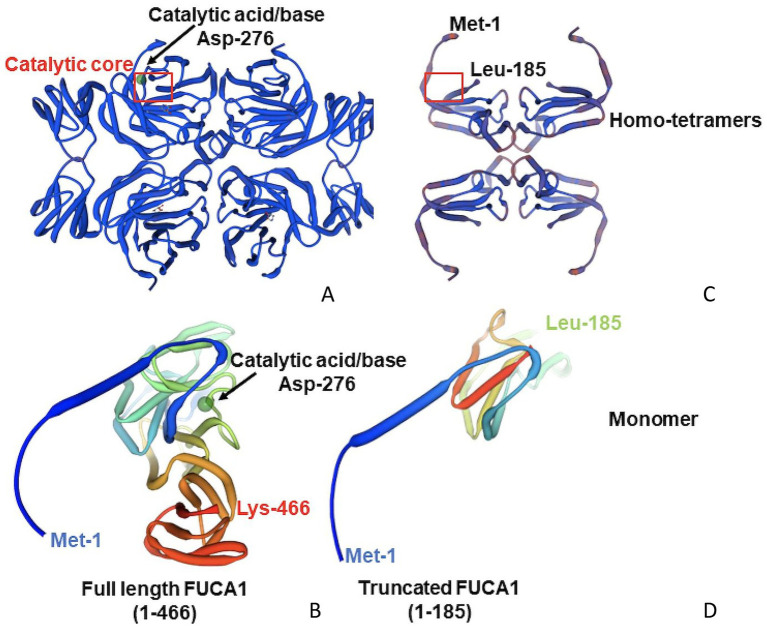
**(A,B)** The catalytic core of FucA1 is located within the α/β-hydrolase domain, where Armstrong et al. identified aspartate-276 as the critical acid/base residue; **(C,D)** c.557T>A (p.Leu186*), introduces a premature stop codon, resulting in a truncated α-L-fucosidase protein that ends at amino acid residue 185.

This variant is completely absent from large population reference databases (gnomAD) and remains entirely unreported in the peer-reviewed medical literature or HGMD registries (2024). While the testing laboratory has registered the variant’s coordinate in ClinVar, this manuscript represents the first formal clinical case report and comprehensive phenotypic description of the p.Leu186* variant in the global literature. This variant introduces a premature stop codon (p.Leu186*), truncating the α-L-fucosidase protein at residue 185 and eliminating the catalytic core within the α/β-hydrolase domain (Figures 3C,D). In silico predictive models indicate that the thymine-to-adenine substitution (c.557 T > A) is expected to trigger nonsense-mediated mRNA decay (NMD) or produce a severely truncated α-L-fucosidase protein lacking essential residues 186–461. The predicted loss of the highly conserved (β/α)₈-barrel structural core, including the critical Asp276 catalytic acid/base residue, is expected to result in profound protein instability and complete loss of enzymatic function. This mechanism is strongly supported by our patient’s complete absence of biochemical α-L-fucosidase activity in whole blood and likely explains the severe neurodegenerative phenotype observed. Furthermore, this novel truncating variant expands the mutational spectrum of FUCA1 and highlights the essential role of the catalytic region in α-L-fucosidase activity. According to ACMG/AMP criteria, this variant is classified as pathogenic based on PVS1 (predicted loss-of-function due to a null variant), PM2 (absence or extreme rarity in population databases), and PP4 (highly specific phenotype consistent with fucosidosis).

### Genotype–phenotype correlation

The correlation of *FUCA1* mutations and clinical phenotypes is poorly defined due to its rarity and heterogeneity. However, truncating mutations, such as nonsense and frameshift variants, are typically associated with severe, early-onset symptoms, and rapid progression (3, 18) as demonstrated in our patient, whose homozygous nonsense variant (p.Leu186*) led to profound enzymatic deficiency and early neurodevelopmental deterioration (see Table 1). Conversely, missense mutations, which may retain residual enzymatic activity, are more frequently linked to attenuated phenotypes with slower progression. Some splice-site variants have been associated with a later-onset disease, characterized by normal early development, rapid clinical decline after 3 years of age (19). These observations highlight the devastating impact of complete enzymatic deficiency.

**Table 1:** Clinical comparison of selected truncating *FUCA1* variants reported in the literature versus the current case.

Exon	DNA alteration (GRCh37)	Protein impact	Geographical/ethnic origin	Core clinical phenotype	Systemic storage manifestations	Reference
Exon 1	c.22C>T	p.Arg8*	Southern Italy	Severe Type I phenotype with early infantile regression, rapid neurodegeneration/decerebration, and death before age 5 years.	Prominent hepatosplenomegaly, severe coarse facial features, and early skeletal abnormalities (dysostosis multiplex).	(3)
Exon 5	c.722delG	p.Gly241Glufs*31	Hispanic (Southwestern United States)	Intermediate Type I/II phenotype with delayed early developmental milestones, loss of speech by age 4 years, and survival into the second decade.	Moderate coarse facial features, progressive angiokeratomas, and absent or mild organomegaly.	(18)
Exon 4	c.557T>A	p.Leu186*	Hispanic (Mexican descent)	Intermediate progressive neurodegenerative phenotype; sat at 14 months, crawled at 16 months, complete motor loss by age 6 years, and severe illness-triggered status dystonicus at age 9 years. Initially misdiagnosed as spastic quadriplegic cerebral palsy.	Mild-to-moderate coarse facial features, localized angiokeratoma-like truncal papules, gibbus deformity, and complete absence of hepatosplenomegaly.	Current study

### Management of fucosidosis

While allogeneic Hematopoietic Stem Cell Transplantation (HSCT) represents the primary therapeutic option capable of halting neurodegenerative progression by supplying donor-derived enzyme across the blood–brain barrier, it was not utilized for this patient. This therapeutic window was missed due to a combination of an initially unremarkable metabolic screening at age two, a prolonged diagnostic odyssey spent outside the U.S. during the peak of the COVID-19 pandemic, and subsequent care fragmentation. By the time his homozygous truncating mutation was discovered at age six, severe, irreversible structural central nervous system changes, profound neurodevelopmental arrest, and advanced skeletal dysplasia were already firmly established. Because the procedural mortality of allogeneic marrow conditioning is exceptionally high, and because transplant cannot reverse established structural neuro-radiological or orthopedic damage, HSCT was deemed clinically inappropriate, limiting his long-term care strategy to intensive multi-specialty supportive management.

HSCT has been proposed for early-stage fucosidosis (20–22) to stabilize neurological decline and increases α-L-fucosidase levels in plasma, white blood cells, and CSF (23). It may improve psychomotor skills, swallowing, and reducing infections (24, 25), however HSCT remains experimental and outcomes are variable once irreversible damage has occured. Enzyme Replacement Therapy (ERT) is in preclinical stages, with intracisternal recombinant α-L-fucosidase showing promising in animals (26), but challenges remain in delivery and long-term efficacy. Gene Therapy using CRISPR-Cas9 is under investigation, no trials have begun and safety concerns persists (27). This case highlights the limitations of current treatments for advanced fucosidosis. This underscores the need for early diagnosis, expanded newborn screening, and accelerated development of disease-modifying therapies.

## Conclusion

This case illustrates the phenotypic heterogeneity of fucosidosis, with progressive neurological symptoms, coarse facial features, and atypical angiokeratoma-like skin lesions but lacking classic systemic features—leading to diagnostic delay. Recurrent respiratory illnesses and HSV-1 infections highlight the risk of opportunistic illness. The identified novel pathogenic variant expands the FUCA1 mutation spectrum and provides valuable insights into genotype–phenotype correlations. Early genetic testing is critical for timely diagnosis, particularly in atypical or incomplete presentations. Mortality often results from respiratory and neurological complications, with many patients dying before age 10 (28). Early HSCT may improve outcomes, while enzyme and gene therapies remain under investigation.

## Data Availability

The datasets presented in this study can be found in online repositories. The names of the repository/repositories and accession number(s) can be found in the article/supplementary material.
